# Supplementary material for: Imaging the neural substrate of trigeminal neuralgia pain using deep learning
Source: Front Hum Neurosci. 2023 May 18;17:1144159. doi: 10.3389/fnhum.2023.1144159 (PMC10232768; doi:10.3389/fnhum.2023.1144159)
Supplement: Supplementary file 1 [file Data_Sheet_1.docx]

Supplementary Material

**Imaging the Neural Substrate of Trigeminal Neuralgia Pain Using Deep Learning**

Yun Liang^1^, Qing Zhao^1^, Zhenhong Hu^1^, Ke Bo^2^, Sreenivasan Meyyappan^3^, John K. Neubert^4*^, Mingzhou Ding^1*^

^1^ J. Crayton Pruitt Family Department of Biomedical Engineering, University of Florida, Gainesville, FL

^2^ Department of Psychological and Brain Sciences, Dartmouth College, Hanover, NH 03755, USA

^3^ Center for Mind and Brain, University of California, Davis, CA

^4^ Department of Orthodontics, University of Florida, Gainesville, FL

*** Correspondence:**

Mingzhou Ding ([mding@bme.ufl.edu](mailto:mding@bme.ufl.edu))

John K. Neubert ([jneubert@dental.ufl.edu](mailto:jneubert@dental.ufl.edu))

# Supplementary Figures and Tables

## Supplementary Figures

**
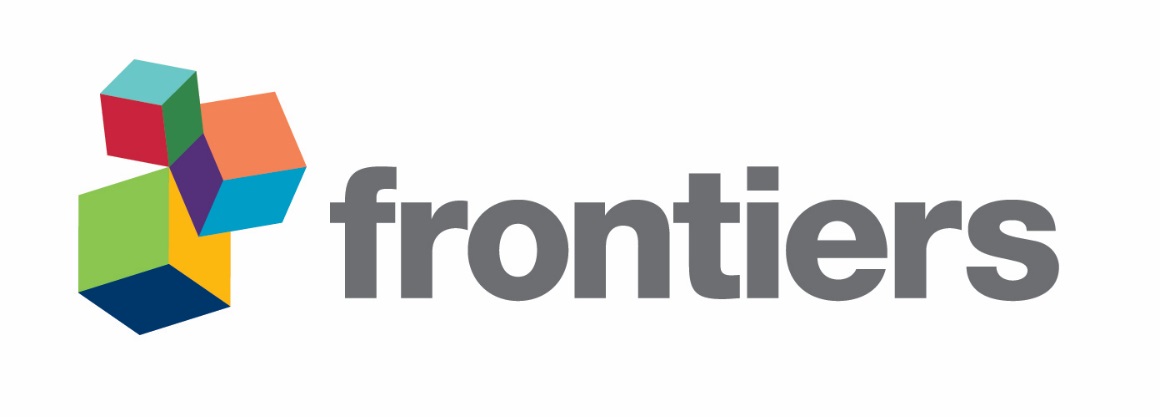
**

**
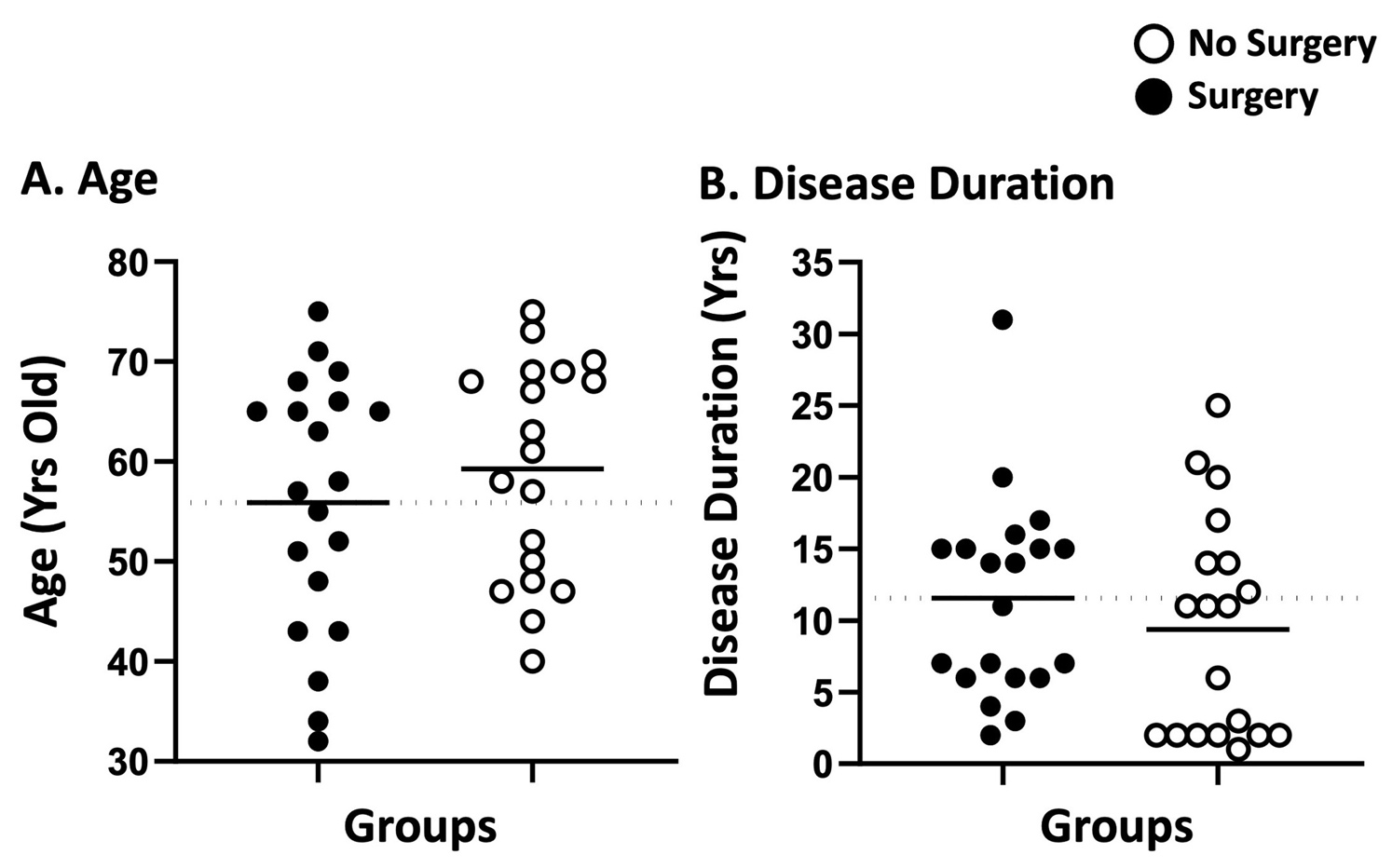
Supplementary Figure 1. Surgery vs. non-surgery subgroup. (A)** Age distribution and **(B)** Disease duration. Surgical and non-surgical groups had a similar age distribution and there was no significant effect from surgical status on disease duration.


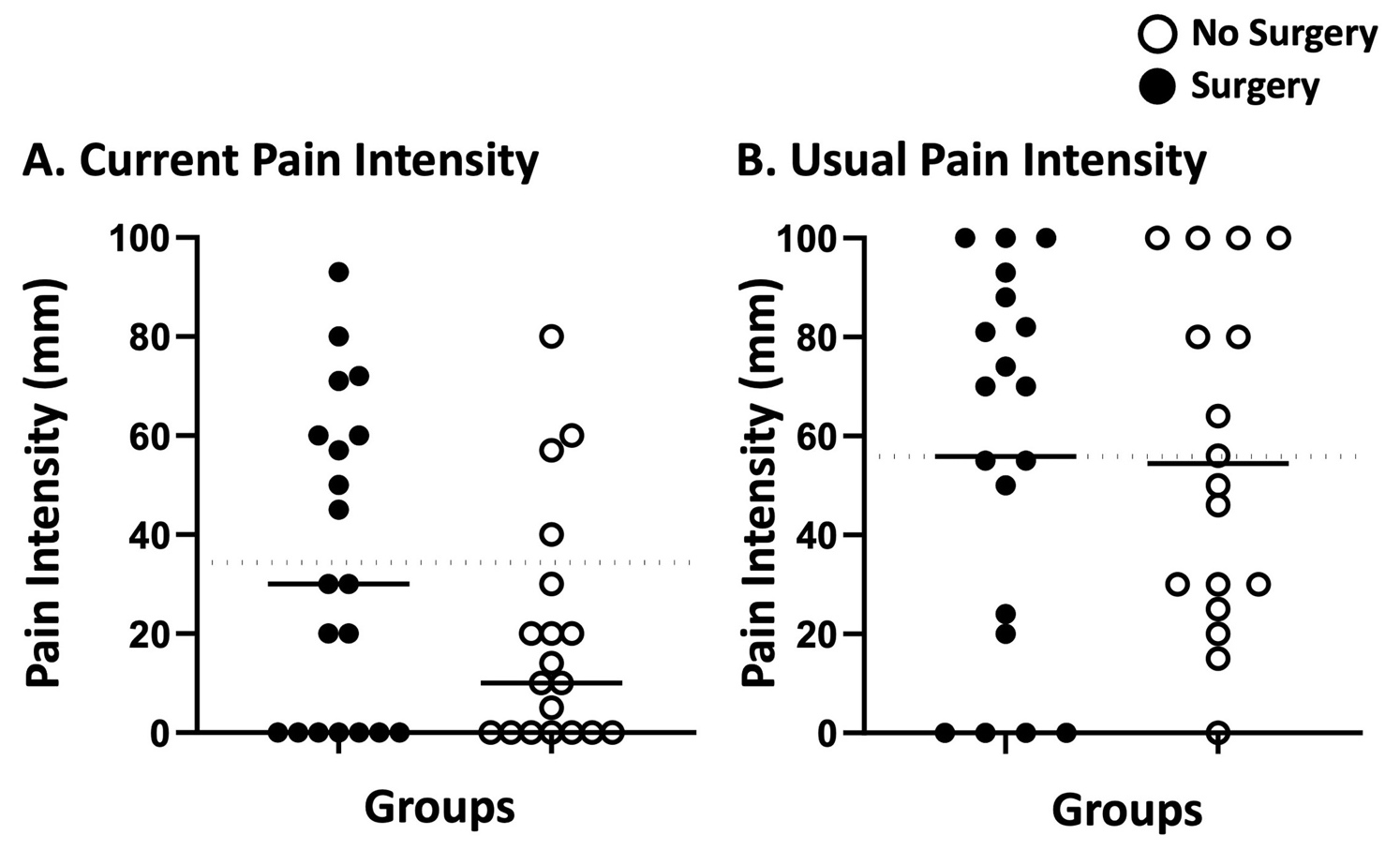
**Supplementary Figure 2. Surgery vs. non-surgery subgroup. (A)** Current pain and **(B)** Usual pain intensity. Pain levels were similar for both surgical and non-surgical groups regarding the pain reported at time of scanning as well as reported usual pain intensity.

**Supplementary Figure 3. Female vs. male subgroup. (A)** Age distribution and **(B)** Disease duration. Male subjects were significantly older (*P=0.0025*, unpaired t-test) as compared to female subjects; however, there was no difference in disease duration between the sexes.

**
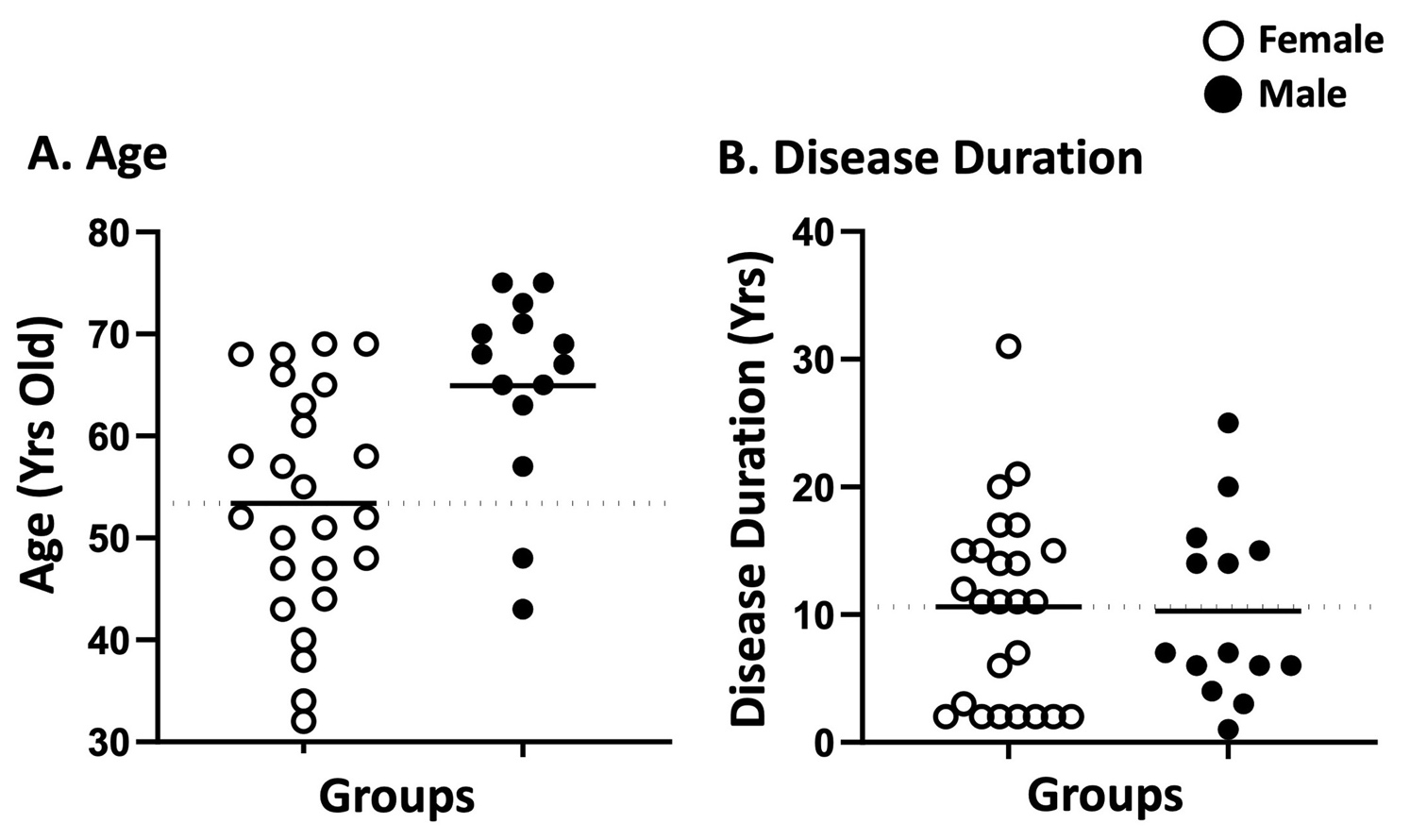
**

**
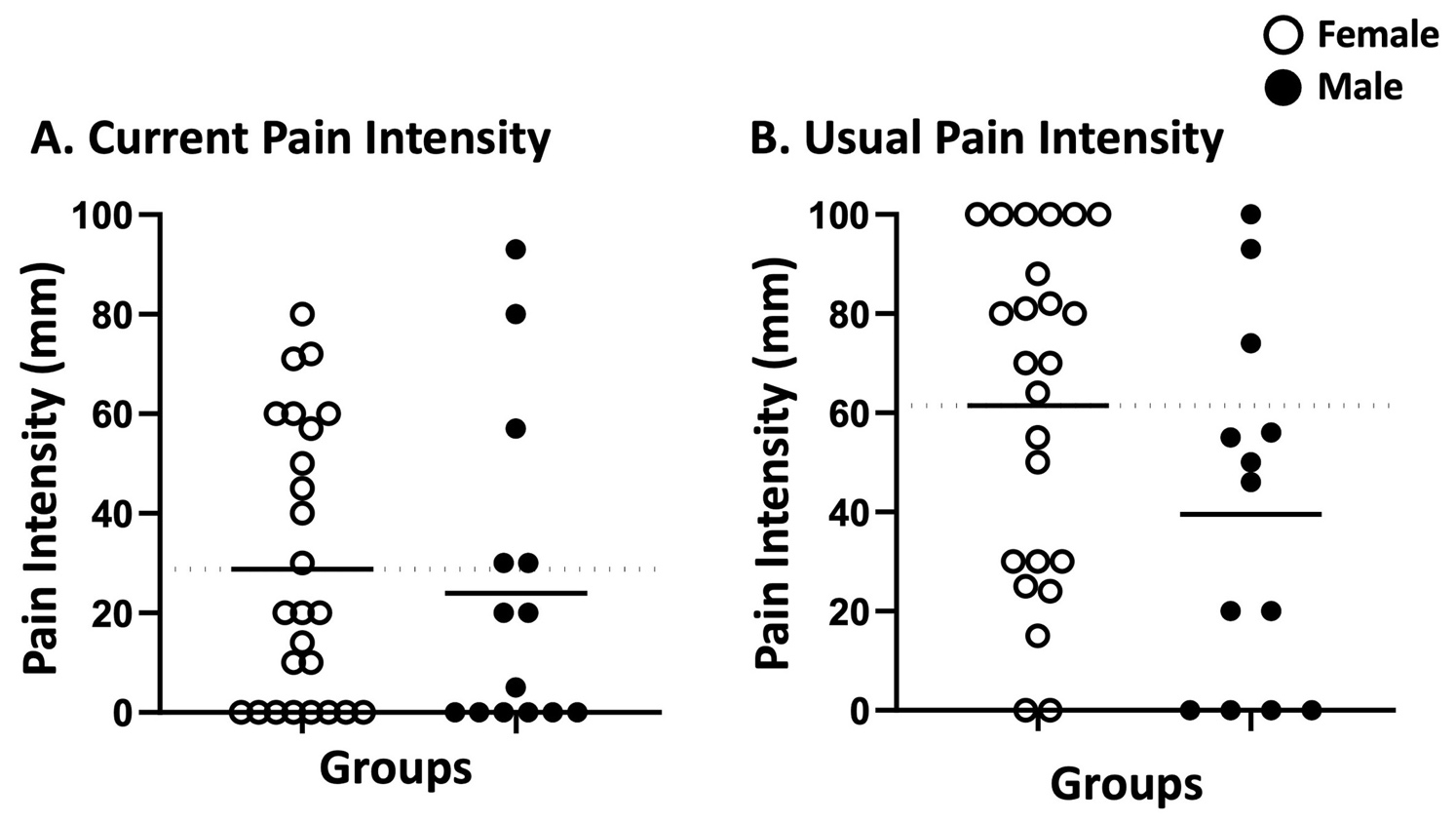
Supplementary Figure 4. Female vs. male subgroup. (A)** Current pain and **(B)** Usual pain intensity. There was no sex difference in either current or usual pain intensity.

## Supplementary Tables

**Supplementary Table 1. Surgery vs. non-surgery subgroup.** Sex ratio, PCS, and BDI-2 information were shown. The BDI-2 score was significantly higher (*P=0.0487*, unpaired t-test) for the surgical subjects, indicating that this group likely suffered from higher depression as compared to subjects that did not have surgery. PCS scores were not significantly different between the two groups.

|  | | **Sex (M: F)** | **PCS score** | **BDI-2 score** |
| --- | --- | --- | --- | --- |
| **Surgery**  (*n=20*) | Mean |  | 26 | 15 |
|  | SD |  | 10 | 9 |
|  | Median |  | 26 | 13 |
|  | Range |  | 9-43 | 1-36 |
|  | Ratio | 9:11 |  |  |
| **No Surgery**  (*n=19*) | Mean |  | 24 | 9 |
|  | SD |  | 15 | 8 |
|  | Median |  | 66 | 6 |
|  | Range |  | 4-52 | 0-27 |
|  | Ratio | 5:14 |  |  |
| **Significance (Surgery vs Non surgery)** | | | *P=0.5237* | ****P=0.0487*** |

**Supplementary Table 2. Surgery vs. non-surgery subgroup.** PASS total and subcategory scores were shown. The PASS total and subscale (Fear, Cognitive, Escape/Avoidance, and Physiological) scores were not significantly different between surgery and non-surgery subjects. This indicates that they exhibited similar levels of pain and anxiety feelings.

|  | | **PASS total score** | **PASS**  **Factor 1: Fear** | **PASS Factor 2: Cognitive** | **PASS**  **Factor 3: Escape/Avoidance** | **PASS**  **Factor 4: Physiological** |
| --- | --- | --- | --- | --- | --- | --- |
| **Surgery**  (*n=20)* | Mean | 90 | 21 | 28 | 26 | 16 |
|  | SD | 27 | 8 | 8 | 7 | 10 |
|  | Median | 87 | 20 | 26 | 27 | 14 |
|  | Range | 50-135 | 10-28 | 16-39 | 10-42 | 0-33 |
| **No Surgery**  (*n=19*) | Mean | 79 | 21 | 25 | 24 | 12 |
|  | SD | 41 | 10 | 11 | 12 | 12 |
|  | Median | 66 | 20 |  | 26 | 9 |
|  | Range | 12-170 | 6-40 |  | 1-43 | 0-50 |
| **Significance**  **(Surgery vs Non surgery)** | | *P=0.3346* | *P=0.8547* | *P=0.4162* | *P=0.4071* | *P=0.2511* |

**Supplementary Table 3. Female vs. male subgroup.** PCS and BDI-2 information were shown. There was no sex difference in the PCS and BDI-2 test scores, indicating that female and male subjects exhibited similar catastrophizing and depression measures.

|  | | **PCS score** | **BDI-2 score** |
| --- | --- | --- | --- |
| **Female**  (*n=25*) | Mean | 26 | 13 |
|  | SD | 13 | 10 |
|  | Median | 29 | 12 |
|  | Range | 4-52 | 0-36 |
| **Male**  (*n=14*) | Mean | 24 | 11 |
|  | SD | 11 | 7 |
|  | Median | 24 | 9 |
|  | Range | 7-36 | 3-23 |
| **Significance (Female vs Male)** | | *P=0.5592* | *P=0.5171* |

**Supplementary Table 4. Female vs. male subgroup.** PASS total and subcategory scores were shown. The PASS total and four subscale scores were not significantly different between males and females.

|  | | **PASS total score** | **PASS Factor 1: Fear** | **PASS Factor 2: Cognitive** | **PASS Factor 3: Escape/Avoidance** | **PASS Factor 4: Physiological** |
| --- | --- | --- | --- | --- | --- | --- |
| **Female**  (*n=25*) | Mean | 87 | 21 | 27 | 26 | 17 |
|  | SD | 38 | 10 | 9 | 10 | 12 |
|  | Median | 85 | 20 | 24 | 26 | 16 |
|  | Range | 12-170 | 6-40 | 5-44 | 1-43 | 0-50 |
| **Male**  (*n=14*) | Mean | 80 | 20 | 27 | 24 | 10 |
|  | SD | 27 | 7 | 9 | 9 | 8 |
|  | Median | 79 | 19 | 26 | 28 | 10 |
|  | Range | 46-128 | 10-33 | 9-40 | 6-35 | 0-29 |
| **Significance (Female vs Male)** | | *P=0.5777* | *P=0.7299* | *P=0.9528* | *P=0.5393* | *P=0.0776* |

**Supplementary Table 5. Detailed structure of the proposed ResNeXt-101.**

| Model | Block | Conv1 | Conv2 | | Conv3 | | Conv4 | | Conv5 | | Average Pooling  SoftMax |
| --- | --- | --- | --- | --- | --- | --- | --- | --- | --- | --- | --- |
|  |  |  | F | N | F | N | F | N | F | N |  |
| ResNeXt-101 | ResNeXt | Conv,7x7x7, 64, stride 2, padding 3 | 128 | 3 | 256 | 4 | 512 | 23 | 1024 | 3 |  |

*F is the number of feature channels, and N is the number of blocks in each layer.

**Supplementary Table 6. Hyperparameter settings for both CNN and GCNN.**

|  | **CNN** | **GCNN** |
| --- | --- | --- |
| Learning rate | 1.0e-3 | 1.0e-3 |
| Epoch | 50 | 50 |
| Optimizer | SGD | SGD |
| Batch size | 2 | 1 |
| Momentum | 0.9 | 0.9 |
| Weight decay | 5.0e-4 | 5.0e-4 |
